# Genetic access to the obligate cyanobacterial endosymbiont *Nostoc azollae* within the *Azolla* fern host

**DOI:** 10.1093/ismeco/ycag209

**Published:** 2026-07-16

**Authors:** Cristina Sarasa-Buisán, Erbil Güngör, Enrique Flores, Peter Lindblad, Sandra Nierzwicki-Bauer, Henriette Schluepmann

**Affiliations:** Instituto de Bioquímica Vegetal y Fotosíntesis, CSIC and Universidad de Sevilla, 41092 Seville, Spain; Biology Department, Utrecht University, 3584 CH Utrecht, The Netherlands; Instituto de Bioquímica Vegetal y Fotosíntesis, CSIC and Universidad de Sevilla, 41092 Seville, Spain; Microbial Chemistry, Department of Chemistry-Ångström, Uppsala University, 75120 Uppsala, Sweden; Department of Biological Sciences and Darrin Fresh Water Institute, Rensselaer Polytechnic Institute, Troy, NY 12180-3590, United States; Biology Department, Utrecht University, 3584 CH Utrecht, The Netherlands

**Keywords:** *Azolla*, conjugation, cyanobacteria, fern, symbiosis

## Abstract

Eukaryote-associated microbes are ubiquitous, but their essential roles in the development and ecology of their host is yet to be fully understood, partly because complex associations cannot be reconstituted and, in many instances, the genetic tools to elucidate those roles are not available. Here, we report the conjugative transfer of DNA into *Nostoc azollae* within three *Azolla* fern hosts. *N. azollae* is a filamentous, N_2_-fixing, heterocyst-forming cyanobacterium which is the predominant obligate endosymbiont of the complex microbial community associated with the floating ferns of the genus *Azolla*. The cyanobiont provides fixed nitrogen to its host, supporting maximum growth rates without any N-fertilizer and making *Azolla* symbioses both ecologically and agriculturally important. Triparental mating protocols and fluorescent reporter detection were optimized for the cyanobiont isolated from the fern, allowing the further demonstration of heterologous gene expression in *N. azollae* driven by several promoters, including some of a CRISPR-associated transposon system. *Azolla* was then treated with a cytokinin hormone to render fern shoot apexes amenable to *in planta* conjugation, permitting DNA transfer to, and gene expression in two distinct developmental stages of *N. azollae* within *Azolla*. These included (i) cells of filaments from the Shoot Apical *Nostoc* colony, the only cyanobacterial stem-cell population vertically transmitted across fern generations, and (ii) cells from differentiated filaments in early formed *Azolla* leaf cavities. Our approach represents a technically groundbreaking advance for the genetic engineering of cyanobacterial endosymbioses that may be useful for other symbiotic systems, opening a way to investigate these important biological entities.

## Introduction

Ferns from the genus *Azolla* and their cyanobiont *Nostoc azollae* form one of the most intimate endophytic symbioses among plant–cyanobacterial associations [1, 2]. *N. azollae* is a filamentous, N_2_-fixing, heterocyst-forming cyanobacterium that transfers to its host ~40% of the N it fixes in heterocysts, a process that occurs within specialized leaf cavities of the fern [3, 4]; it is a vertically inherited obligate symbiont that exhibits extensive genome erosion [2, 5]. This vertical inheritance is mediated by a specific and persistent population of *N. azollae* located in the shoot apical meristem of the fern, known as the Shoot Apical *Nostoc* Colony (SANC), which is characterized by filaments with absence of heterocysts and narrowed cells that serve as the inoculum for newly developing leaves and reproductive structures [6, 7]. *N. azollae* is supported at least partially by sugar received from its host [8, 9]. This mutualism enables *Azolla* to thrive in nitrogen-poor environments with traditional uses as a biofertilizer in rice crops. Today, *Azolla* cultivation is also promising for a high-protein food and feed, a biofuel, bioremediation, greenhouse gas mitigation, and pest control [10–16]. However, its adoption will require that biomass yields are stabilized, which depends in part on the development of strains that are resistant to common biotic stressors.

The *Azolla*–*Nostoc* symbiosis remains genetically intractable. On the plant side, progress has been made through protoplast induction, sexual crossing, cryopreservation, (meta)genome sequencing, and transcriptomic analyses [17–19], but stable transformation of *Azolla* has yet to be achieved. On the cyanobiont side, the obligate endosymbiotic nature of *N. azollae* restricts experimental approaches to *in planta* DNA delivery methods. Moreover, the *N. azollae* genome has lost most genes involved in natural competence (see Table S1 from Wendt & Pakrasi, 2019 [20]), limiting methods of DNA delivery to active DNA mobilization such as bacterial conjugation. Therefore, the question arises, after ~100-Myr of evolution of *N. azollae* within *Azolla* [21, 22], can *N. azollae* still undergo conjugation?

In this work, we assess whether an *Escherichia coli*–mediated conjugation with an eYFP-monitoring system, optimized in model cyanobacteria, could be adapted to *N. azollae* firstly outside the fern and then *in planta*, specifically targeting the Shoot Apical Nostoc Colony (SANC), the *N. azollae* population capable of being inherited through *Azolla* vegetative and sexual cycles [6, 7]. Additionally, we show the delivery of a large genome editing tool, the CRISPR-associated transposon (CAST) system. CAST is a powerful tool, enabling precise, large DNA insertions independent from host repair pathways [23], which has been validated in a model free-living, heterocyst-forming cyanobacterium, *Anabaena* sp. PCC 7120, for YFP-tagged deletions [24].

## Materials and methods

### *Azolla* fern and cyanobacterial cultures

Three different species of *Azolla* were used, *A. filiculoides* strain Galgenwaard [22], *A. anzali* [19], and a freshly isolated *Azolla* strain sampled from Doñana National Park (Huelva, Spain), hereafter referred to as “*A. filiculoides* strain Doñana,” whose originating population was first detected in 2001 and identified as *A. filiculoides* [25]. *Azolla* sporophytes were cultivated in Standard *Azolla* Medium, a variation of IRRI2 medium [19], at 20°C under a 16-h light/8-h dark cycle, in which the light period (100 μmol m^−2^ s^−1^, LED-tube 4000K) was supplemented with Far-Red light from an APEXstrip Bundle 730 nm – 16 W system (Crescience). The cyanobacterium *Anabaena* sp. strain PCC 7120 (hereafter *Anabaena*) was cultured photoautotrophically in BG11 or BG11_0_ (BG11 without NaNO₃) medium at 30°C, under constant light (30 μmol photons m^−2^ s^−1^, LED-tube 4000 K) unless otherwise stated. BG11 medium was as described [26] with ferric ammonium citrate substituted by ferric citrate. Liquid cultures were maintained in Erlenmeyer flasks and shaken. For cyanobacterial solid cultures, 1% (w/v) agar (Bacto-Agar, Difco) was added. When required, growth medium was supplemented with the indicated concentrations of antibiotics. All strains used in this work are summarized in Table S1.

### Plasmid constructions and cloning procedures

All plasmids generated in this work were developed using Golden Gate cloning and are summarized in Table S1. Level 0 plasmids were built with previous parts [24] such as P*_glnA_* and PCR-adapting new individual components obtained from CyanoGate [27] or MoClo Plant level 0 plasmids [28]. Level 1 plasmids were assembled by digestion with *Bsa*I and ligation. To obtain level T plasmids, level 1 inserts were assembled into the backbone vector pCAT.000 (conjugative and replicative vector harboring the RSF1010 origin of replication from the Cyanogate kit), together with an end-linker (L), by *Bpi*I digestion and ligation to create either the pCAST plasmids harboring the CAST system or the vectors designed for promoter testing (pLX). Detailed information of the plasmids and key characteristics of the tested promoters can be found in Table S1. For pCAST plasmids, the desired sgRNA was cloned with one *Lgu*I reaction either into Level 1 pAzU1.3 plasmid as described previously [24] or directly into the final pCAST empty plasmids named pCAST01 (erythromycin resistance [Em^R^]) and pCAST02 (spectinomycin/streptomycin resistance [SpSm^R^]). Sequences of oligonucleotides used in this work are summarized in Table S2. Final pCAST vectors ready to clone any sgRNA by one *Lgu*I reaction have been deposited in Addgene. Reactions for Golden Gate assembly of Level 1 and Level T plasmids and sgRNA oligonucleotide annealing are described in Supporting information. Restriction enzymes and T4-DNA ligase were from ThermoFisher. Plasmids were introduced in *E. coli* DH5α and HB101 by heat shock [29]. Putative positive colonies were selected using appropriate antibiotics and blue–white screening [30]. Positive colonies were confirmed either by PCR with Taq polymerase using specific primers or by plasmid isolation and restriction. All plasmid sequences were finally checked by Nanopore sequencing (Plasmidsaurus).

### Triparental mating procedure for *Anabaena* and *N. azollae ex planta*

Triparental mating was performed following the procedure of Elhai and Wolk [31]. In addition to the recipient cyanobacterium, this procedure used (i) *E. coli* ED8654 harboring pRL443, which provides the conjugation machinery; and (ii) *E. coli* HB101 carrying the “helper” plasmid pRL623, which bears a *mob* (ColK) gene and three methylase genes to protect the cargo plasmid from the native restriction enzymes *Ava*I, *Ava*II, and *Ava*III in *Anabaena* [32], and the cargo plasmid, which is the construct intended to be delivered into the cyanobacterial recipient. Because *N. azollae* appears to bear restriction/methylation systems different to those of *Anabaena* [33], the inclusion of Ava methylases may not help in conjugation into *N. azollae* but, in any case, it is not expected to hamper this process. Overnight precultures of both *E. coli* strains were used to initiate fresh cultures by inoculating 10 ml of Luria–Bertani (LB) medium (without antibiotics) with 250 μl in the case of ED8654 preculture and 350 μl for the HB101 preculture. Refreshed cultures were incubated for 2.5 h at 37°C with shaking to reach exponential growth. Cells from donor and helper cultures were then transferred to round-bottom tubes and gently centrifuged at 1600 × *g* for 3 min at room temperature (RT). Supernatants were removed, and pellets were resuspended in remaining LB medium and mixed by gently tapping the tubes. Donor and helper cells were incubated statically at 25°C for 2–4 h and then mixed with filamentous cyanobacteria: an amount of cells containing 10 μg of chlorophyll *a* (Chl) for *Anabaena* and 20 μg Chl of *Azolla* juice containing *N. azollae* obtained as previously described [9] using BG11_0_ buffered with 10 mM TES (N-tris(hydroxymethyl)methyl-2-aminoethanesulfonic acid), pH 7.5, as extraction medium to prevent possible pH variations due to release of *Azolla* trichome acidic content. Chl was determined in methanolic extracts of the cells as previously .described [34]. The higher amount of *N. azollae* cells compared to *Anabaena* was used to compensate for potential underestimation of cyanobacterial Chl due to remanent plant debris Chl. The cyanobacteria–*E. coli* mixture was gently spread onto nitrocellulose filters (Immobilon, Millipore REF: HAFT08550) placed on plates containing a solidified mixture of 95% BG11 or BG11_0_ (for *N. azollae* conjugation experiments with *Anabaena* used as control) medium and 5% LB. After 24 h, filters were transferred to BG11 or BG11_0_ medium, respectively. For selection in *Anabaena*, filters were transferred after 24–48 h to plates of BG11 medium supplemented with appropriate antibiotics (25 μg/ml neomycin, 5 μg/ml spectinomycin sulfate and 5 μg/ml streptomycin sulfate, or 10 μg/ml erythromycin). In *N. azollae ex planta* conjugation experiments, conjugal filters were incubated in darkness during the first 16–24 h of mating and then maintained under a 12-h light/12-h dark photoperiod; white-light intensity, 30 μmol photons m^−2^ s^−1^.

### Preparation of *Azolla* for *in planta* conjugation of the obligate symbiont *N. azollae*

A detailed scheme of the “*in planta*” *N. azollae* conjugation is shown in Fig. S1A. Access to the genetic target in the Shoot Apical *Nostoc* Colony (SANC) was performed by exposing the Shoot Apical Meristems (SAMs) of *Azolla* to cytokinin (BAP; 6-benzylaminopurine) treatment. Fresh growing *Azolla* sporophyte cultures were used to set up cultures of 3-finger plantlets in 50 ml of fresh medium as starting point. After 1 week, they were sub-cultured in fresh medium with and without 0.5 mg/L BAP (for *A. filiculoides* strain Galgenwaard) or 1 mg/L BAP (for *A. anzali* and *A. filiculoides* strain Doñana). Hormone treatment was performed for 24 h based on experiments to determine the optimal hormone treatment protocol (see Supp. Fig. S2A). To remove BAP, plants were rinsed by vigorous swirling for 5 s in distilled water and transferred to fresh hormone-free culture medium. After 10 days, fronds started developing open meristems and were used for *in situ* triparental conjugation. The opening of *Azolla* apexes after hormone treatment was followed using a stereo microscope Leica M205 C or a fluorescence stereo microscope Zeiss-AxioZoom v16. Helicon focus software was used to generate Z-stack image reconstructions.

### Preparation and inoculation of *E. coli* conjugative mix for *in planta* conjugation

Two approaches, “classical” and “short” were used for the preparation of the *E. coli* conjugative mix as depicted in Table S3. The “classical” protocol was followed by using the *E. coli* conjugative mix prepared as mentioned above for *Anabaena*. After the biparental incubation, the *E. coli* mix was spotted onto an LB plate in 200 μl drops and allowed to dry under a bacteriological hood at RT before use. Alternatively, after the drying step, the biparental mix plate was kept overnight (O/N) at 20°C to be used the following day to reduce processing time (see “classical - O/N” in Table S3). The “short” protocol consisted of preparing the *E. coli* conjugative mix using directly the overnight *E. coli* cultures instead of a refreshed exponential phase *E. coli* culture, as this showed little difference in conjugation efficiency [35]. After gentle washing with fresh LB medium to get rid of antibiotics, *E. coli* cultures were mixed and 200 μl drops of the mix were placed on LB plates and allowed to dry under a bacteriological hood at RT, followed by incubation at 25°C for 3–4 h to obtain the *E. coli* conjugative “cream”. After evaluation, the “short” protocol was adopted as the standard protocol in this study, as it made it possible to inoculate more plants within the same day.

To inoculate the *Azolla* apex with the *E. coli* conjugative “cream,” two glass microneedles (Glass replacement 1.14 mm 3.5″ [(WPI]) were built using the Next Generation Micropipette Puller P-1000 (Sutter instrument) with the following parameters: program 2 at ramp 494 (Fig. S1B). Conjugative *E. coli* “cream” was carefully placed onto exposed SAMs that showed the presence of *N. azollae* dark-green spots from the SANC. The LB plate containing the *E. coli* “cream” drop was kept closed between inoculations to prevent over-drying. The inoculum was gently “massaged” onto the SAM. Each frond, containing two to three treated apexes, was then carefully placed into a well of a 24-well plate containing 1 ml of liquid standard *Azolla* medium. Apex inoculation was performed on actively growing plants typically within 2–3 h before the onset of the dark phase of the diel cycle. Processing 24 fronds required between 2 and 3 h; therefore, inoculations often extended into the early dark phase. Positive conjugation events were also obtained in exploratory assays in which inoculations were performed up to 2 h into the dark phase. Other time points were not selected due to practical constraints of the experimental setup. Following inoculation, fronds were immediately subjected to controlled incubation conditions. After several trials (Table S3), the following conditions were selected to simplify the protocol and maximize standardization. For the first 24 h, plates were incubated in the dark at 30°C, followed by an additional 2 days at 30°C under a 16-h light/8-h dark cycle; white-light intensity, 30 μmol photons m^−2^ s^−1^. For longer incubation times, *Azolla* fronds were placed in fresh growth medium and transferred to regular *Azolla* growth conditions (20°C under a 16-h light/8-h dark cycle; white-light intensity, 100 μmol photons m^−2^ s^−1^ supplemented with Far-Red light).

### Confocal microscopy and monitoring of conjugation events

Cyanobacterial conjugation was monitored through eYFP detection by confocal microscopy. For *Anabaena* conjugation and *ex planta* conjugation with *N. azollae*, a scrape of cells from a conjugation filter was streaked on a plate of BG11_0_ medium, then cut out of the agar and covered with a coverslip for visualization. For screening of *in planta* conjugation, *Azolla* apexes were dissected under a Leica M205 C Stereo Microscope and gently squashed between two coverslips in the presence of 20 μl of 60% glycerol to avoid desiccation. Images were collected with an Olympus FLUOVIEW FV3000 confocal laser-scanning microscope equipped with a UPlanApo 40 × 1.5 NA oil immersion objective. The eYFP was excited using a 488 nm laser at 2% power, with irradiation from an argon ion laser. The fluorescence of eYFP was visualized with a window of 500–540 nm, and the autofluorescence from the natural pigments of cyanobacterial cells was collected using a window of 660–700 nm. ImageJ-FIJI [36]/Olympus software was used to process images.

The conjugation success was followed using confocal microscopy at the selected time points after conjugation. To quantify conjugation events in *N. azollae in planta* experiments, consecutive eYFP expressing cells were considered as an individual conjugation event followed by cell division. Lambda scans, routinely performed to discern eYFP signal from cyanobacterial autofluorescence signal, were recorded over the 500–700 nm spectral window following excitation with a 488 nm laser at 2% power. The average fluorescence intensity within the region of interest was plotted as a function of wavelength.

## Results

### Tracing early conjugation events in cyanobacteria

To assess the feasibility of conjugative DNA transfer in the obligate cyanobiont *N. azollae*, we developed a fluorescence-based methodology to monitor early DNA transfer events in cyanobacteria using the *Anabaena* model. Additionally, we studied whether early conjugation events could be detected using, as cargo, a plasmid carrying the sgRNA-guided transposition CAST system [24]. To enable reliable monitoring of conjugation events, we constructed an RSF1010-based plasmid containing a fluorescence reporter (enhanced Yellow Fluorescent Protein [eYFP]) driven by the strong synthetic *E. coli* promoter P_*trc*1O_ [27, 37]. The resulting fluorescent cassette was tested either as an independent construct within the replicative backbone pCAT.000 (generating plasmid pL0) or by replacing the *P_rnpB_–*eYFP cassette in a previously described CAST plasmid [24] yielding updated versions of CAST plasmids (referred to as pCAST01 [Em^R^] and pCAST02 [Sp^R^Sm^R^]) ready to clone any sgRNA by one single Golden Gate reaction with *LguI* (Table S1).

**Figure 1:**
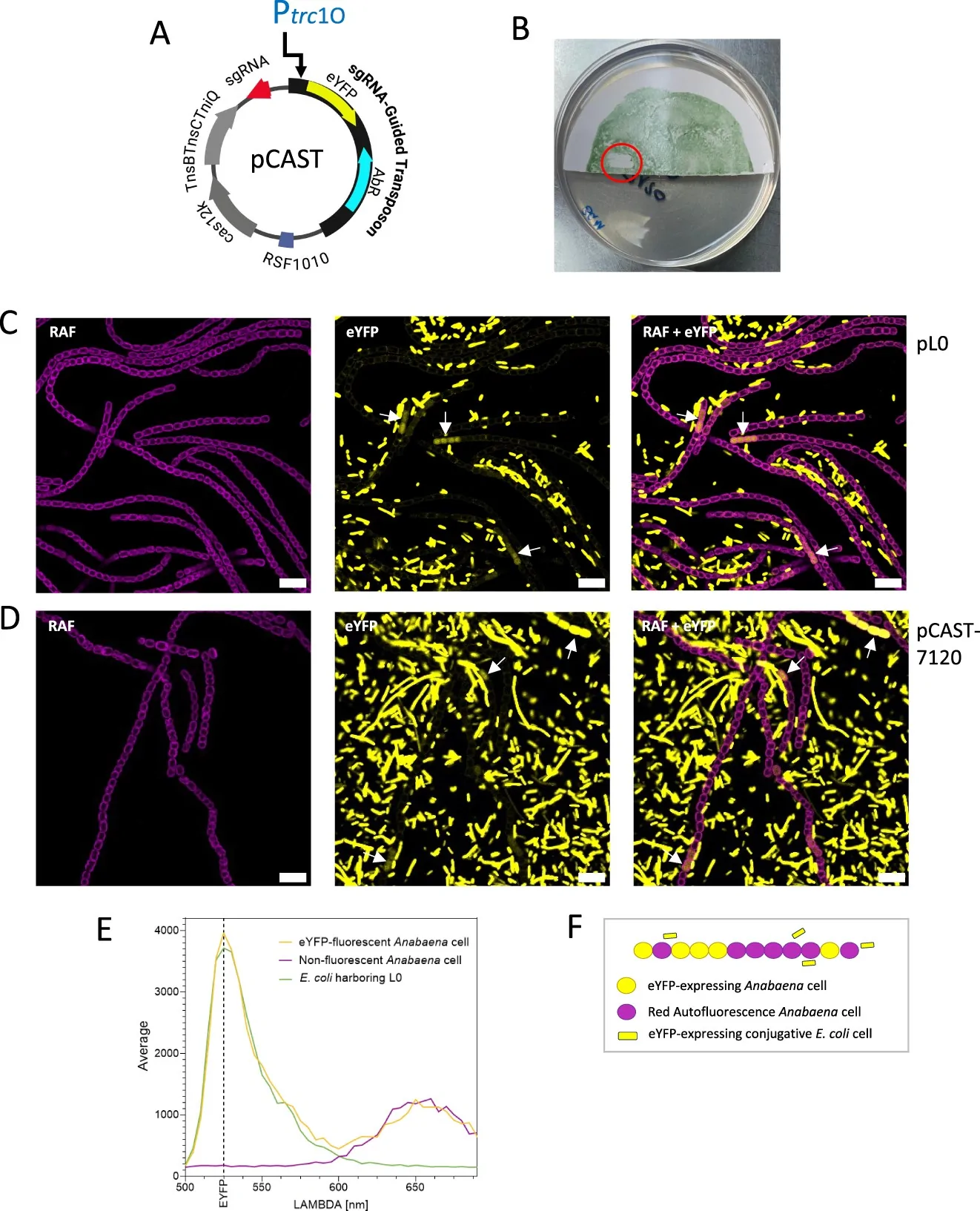
Tracing early conjugation events in *Anabaena* sp. PCC 7120 by confocal microscopy. (A) Schematic representation of the CAST plasmid pCAST-7120, featuring the P*_trc_*_1O_ promoter for eYFP expression. (B) Conjugation plate of *Anabaena* 48 h after conjugation highlighting in red the scrap taken 21 h after conjugation. Part of the conjugation plate was scraped up and re-streaked in a BG11_0_ medium agar plate for visualization. (C, D) Confocal microscopy images displaying conjugation events at Day 2 after conjugation with the control plasmid pL0 (C) and the pCAST-7120 plasmid including a sgRNA targeting *nifK* (D). Some conjugation events are marked with a white arrow. Note the presence of conjugative *E. coli* cells harboring cargo plasmids and expressing the eYFP. The conjugation (triparental mating) was performed as described in Materials and Methods. Red auto fluorescence (RAF); enhanced Yellow Fluorescent Protein (eYFP). Images are representative of at least five independent experiments. Scale bar, 10 μm. (E) Example of lambda scan determination of specific eYFP signal in *E. coli* donor cells and *Anabaena* exconjugants. (F) Scheme depicting the different cells observed, expressing or not expressing the eYFP.

Conjugation events of *Anabaena* receiving pL0 and pCAST02 targeting the *nifK* gene, hereafter referred as pCAST-7120 (Fig. 1A), were monitored 21- and 48-h post-conjugation, as well as after 3 days of antibiotic selection. A scrape of cells from the conjugation plate (Fig. 1B) was transferred to a plate of BG11_0_ medium (which lacks combined nitrogen) and visualized using confocal microscopy. Conjugation events were detected in *Anabaena* as early as 48 hours post-mating, as eYFP signal localized to the cytoplasm of the exconjugants with either pL0 (Fig. 1C) or pCAST-7120 (Fig. 1D). At all observation time points, residual *E. coli* donor cells carrying pL0 or pCAST-7120 were also observed as ~1-μm eYFP-expressing rods or as chain-like aggregates (characteristic of biofilms [38]), which were readily distinguishable from cyanobacterial cells. Conjugation events were verified by Lambda scans (500–700 nm), in which the fluorescence spectra of *Anabaena* exconjugants and controls were analyzed (Fig. 1E). A characteristic emission peak at 525 nm in both *E. coli* donor cells and cyanobacterial exconjugants confirmed the presence of the eYFP. Additionally, the scans verified that *Anabaena* cells displayed their typical autofluorescence derived from cyanobacterial pigments. This procedure was later critical to distinguish true eYFP fluorescence from background cyanobacterial autofluorescence and *Azolla* tissue fluorescence.

A spatial pattern of *Anabaena* exconjugant cells at early stages was observed and suggests that paired adjacent fluorescent cells may represent a single conjugation event followed by cell division, whereas noncontiguous fluorescent cells within the same filament are likely to represent multiple independent conjugation events in that filament (Fig. 1 and Fig. S3). Therefore, this criterion was used for subsequent quantifications of conjugation events. Noteworthy, several noncontiguous exconjugant cyanobacterial cells could display different levels of eYFP fluorescence intensity with either pL0 and pCAST-7120 (Fig. 1 and Fig. S3). This may result from variations in plasmid copy number, as has been observed previously with replicative plasmids in filamentous cyanobacteria [39], or, in the case of pCAST-7120, from the sum of eYFP expression from the replicative plasmid and transposon insertion events into the chromosome that may have already taken place. Because low fluorescence intensity may hinder early detection, this implies that observed conjugation events may be underestimated. Consequently, it is possible that conjugation had already occurred at earlier time points but remained undetectable at 21 h. Modifications in the standard *Anabaena* conjugation protocol (Materials and Methods), such as incubation during the first 24 h in darkness, extending the *E. coli* mating time (prior to mixing with *Anabaena*) from 2 to 4 h, or the use of the Em^R^ pCAST01 version as the backbone (see pCSCSBT22 in Table S1), were tested to explore conditions that might be useful for subsequent *in planta* applications. However, these conditions did not result in an earlier detection.

### Isolated *N. azollae* filaments can be conjugated *ex planta*

**Figure 2:**
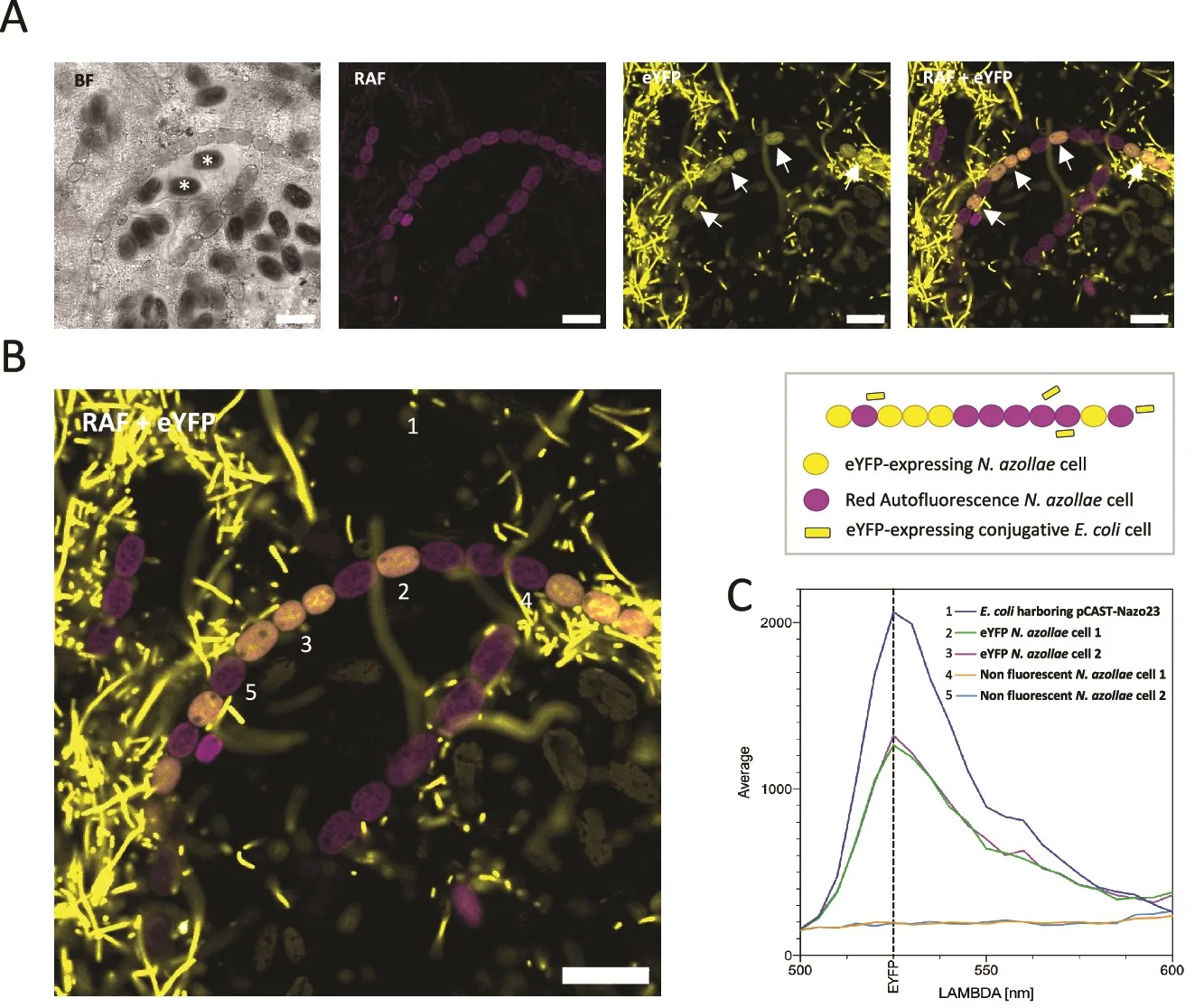
Conjugation events detected through eYFP expression in *N. azollae* isolated from *A. filiculoides*. Conjugation of *N. azollae* cells extracted from *A. filiculoides* strain Galgenwaard was performed and analyzed as described in Materials and Methods. (A) Confocal imaging of *N. azollae* conjugation events in four detection channels: BF (brightfield), RAF (red autofluorescence, eYFP, and merged eYFP + RAF. Several conjugation events of *N. azollae* cells are marked with a white arrow. Possible eukaryotic algae, which were likely present on the surface of the fern, are shown with (*). (B) Enlarged view of the RAF + eYFP fluorescence channel. (C) Average fluorescence emission spectra corresponding to the numbered cells in panel (B). Note fluorescent eYFP *N. azollae* cells showing the same characteristic eYFP emission peak as *E. coli* harboring the eYFP expressing plasmid pCAST-Nazo23. Scale bar, 15 μm. A scheme depicting the different cells observed, expressing or not expressing the eYFP, is also included.

A conjugation protocol “*ex planta*” was established using *N. azollae* filaments extracted from fronds of *A. filiculoides* strain Galgenwaard [5] (Fig. S4), a sample called hereafter *Azolla* juice [9]. The viability for several days of *N. azollae* filaments present in the *Azolla* juice was demonstrated previously [9]. An adapted triparental mating protocol (Fig. S1A) was used with *E. coli* donor strains carrying either the control plasmid pL0 or the pCAST01-based pCAST-Nazo plasmids (pCAST-Nazo23, encoding an sgRNA targeting *nifK*; pCAST-Nazo24, encoding an sgRNA targeting the nonfunctional pseudogene *narB*) (Tables S1 and S2). This yielded detectable conjugation events in *N. azollae* (Fig. 2 and Fig. S5, respectively), with eYFP fluorescence signals matching those of *E. coli* controls in Lambda scan analyses (Fig. 2C). Overall, exconjugants were detected within a time frame of 2–5 days after mating without antibiotic selection, which corresponds with the timing observed for the *Anabaena* control (Fig. 1).

In several cases, eYFP-positive cells could be detected within nonadjacent cells in a single filament, suggesting independent conjugation events. In addition, contiguous eYFP-expressing cells were frequently found, consistent with conjugation followed by cell division (Fig. 2B). In a few cases, eYFP-positive vegetative cells were detected in filaments containing heterocysts (Fig. S5), which reflects that the *Azolla* juice harbors filaments in different developmental states, from leaf pockets (presence of heterocysts and larger cells) or from meristematic regions corresponding to the SANC population (narrowed cells and absence of heterocysts [9]). Nonetheless, overall, conjugation events were frequently detected in short filaments or paired cells (Fig. S6), consistent with filament fragmentation observed in the sample due to the mechanical manipulation of the extraction protocol [9] (Fig. S4). After Day 5 following conjugation, extracted cyanobacterial filaments subjected to conjugation showed signs of reduced viability and reached a near-complete disintegration. Under these circumstances, providing a normalized or comparative conjugation efficiency metric is not feasible, as the progressive reduced viability of extracted *N. azollae* likely limits both transconjugant recovery and reporter detection.

To assess whether this protocol could be used with another host species, we carried out the conjugation assay with *N. azollae* filaments isolated from an *Azolla* line considered to be very robust, *A. anzali* [19]. The same plasmid constructs that had been validated in *A. filiculoides* strain Galgenwaard were used for *A. anzali*. In this case, eYFP-positive cells could also be detected (Fig. S7), showing that conjugation can be achieved in *N. azollae* from different *Azolla* host species.

Together, these findings indicate that *N. azollae,* from two independent *Azolla* host species, have retained the functional traits required to receive DNA by conjugation despite its long-term obligate symbiotic lifestyle. Moreover, the results show that plasmids carrying the RSF1010 origin of replication and a P_*trc*1O_-driven reporter are functional for heterologous gene expression in *N. azollae*, and that the ~16 kb pCAST derived constructs can be replicated and maintained in *N. azollae*, which will be necessary for the development of CAST-based tools in this symbiont.

### *In planta* conjugation of *N. azollae*

**Figure 3:**
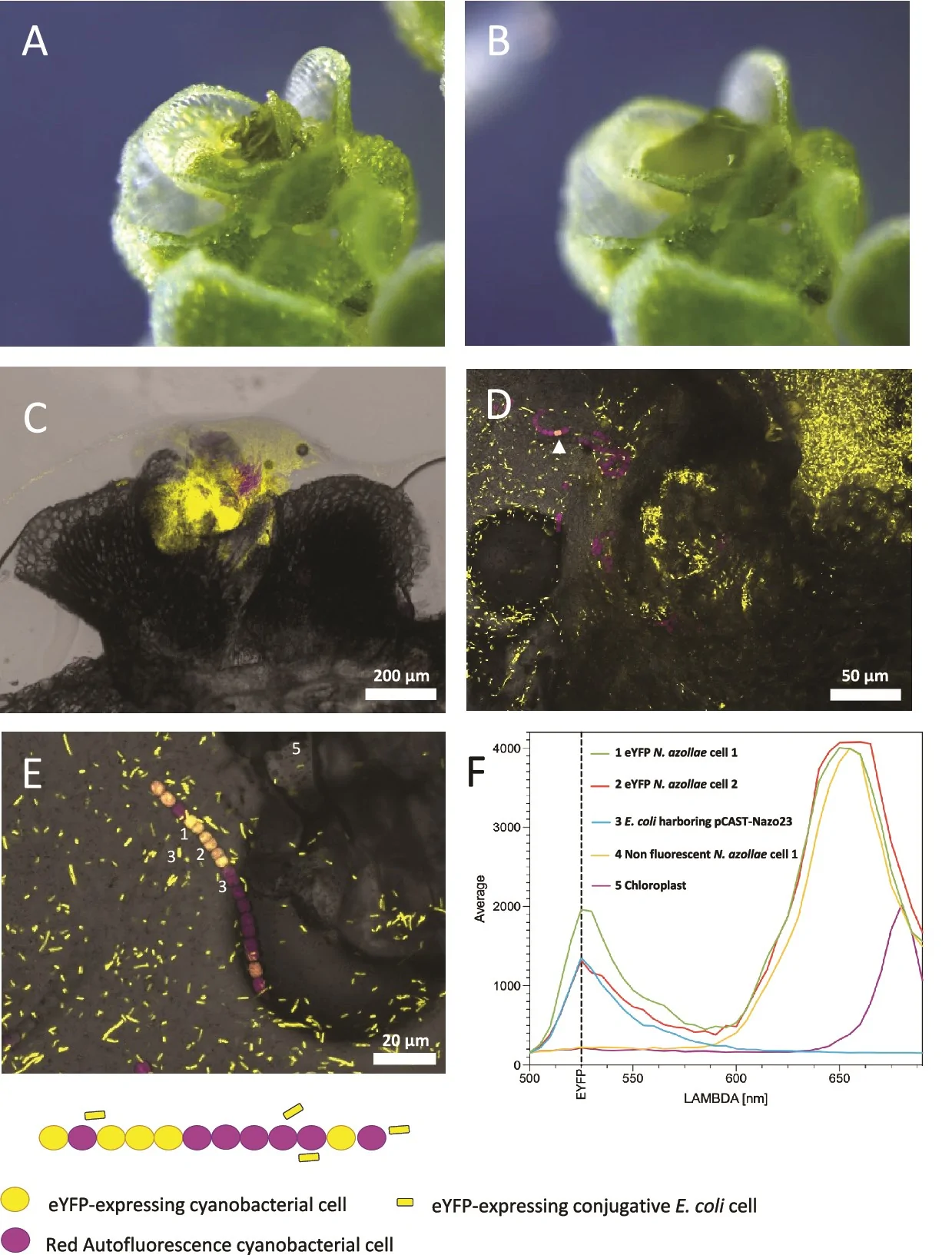
Conjugation of *N. azollae in planta*. (A) Apex of *A. anzali* showing exposed meristem after treatment with 1 μg/ml BAP for 7 days, followed by 13 days without hormones. (B) Apex from “A” immediately after the application of concentrated conjugative *E. coli* suspension with microneedles. (C) Example of inoculated apex of *Azolla* observed by confocal microscopy 24 h postinoculation. (D) Evidence of a single conjugation event of *N. azollae* in *A. anzali* 5 days postinoculation. Confocal images composed by three merged channels: BF, Brightfield; RAF, Red autofluorescence, and eYFP channel. (E) Z stack detail of *N. azollae* filament showing three to four conjugation events. Average fluorescence emission spectra corresponding to the numbered spots from a single focal panel of micrograph from (E) panel. Conjugation was performed with *E. coli* harboring pCAST-Nazo23. (F) A scheme depicting the different cells observed, expressing or not expressing the eYFP, is also included.

Genetic manipulation of *N. azollae* that is transmitted to the developing host organs including the leaves and reproductive structures (and therefore to the next plant generation), specifically requires that conjugative *E. coli* targets the distinct *N. azollae* population called SANC “Shoot Apical *Nostoc* Colony”, which resides within the shoot apex, just above the Shoot Apical Meristem (SAM) of *Azolla* (see Figure 1 in Dijkhuizen *et al*. 2021 [19]). The SANC can be considered an *N. azollae* “stem-cell” colony that colonizes newly formed leaves and reproductive organs as they form [1, 40] (Fig. 3A). The SAM is normally covered by developing leaf initials such that the SANC is inaccessible to *E. coli* (Fig. S8A). To access the SANC, it was necessary to expose the SAM, which was achieved by treatment with the cytokinin 6-benzylaminopurine (BAP) (Figs S2 and S8). Initial experiments with *A. filiculoides* strain Galgenwaard showed that BAP treatment severely weakened the plants, making the tissues more fragile and prone to fragmentation during handling. This fragility often appeared to compromise the health of the apexes during initial conjugation assays, as evidenced during short-term monitoring (48–72 h) in comparison with *A. anzali*. As *A. anzali* did not show this BAP-induced fragility, it was selected for subsequent experiments as a more robust alternative, owing likely to its faster growth under laboratory conditions. SAM exposure was induced with BAP (Fig. S2B), and 24 h of exposure to hormone was sufficient to elicit open apexes detectable after 10 days (shown in Fig. S8). Longer incubation times did not increase the number of open apexes, suggesting that the response saturates within 24 h. The closed SAM phenotype with outgrowing meristems was restored ~1 month post-treatment onward (Fig. S2C).

To further characterize SAM structure and SANC properties, a staining procedure using the fluorescent DNA-binding dye 4′,6-diamidino-2-phenylindole (DAPI) was performed (Fig. S9). The results suggested that it was possible to manually deliver a payload (exogenous material introduced for experimental purposes) to the SANC and helped us to determine optimal imaging conditions for *in planta* confocal microscopy. Once the SAMs were exposed (Fig. 3A), the meristems were “massaged” using glass-made microneedles to facilitate application of a cream-consistency conjugative mix of *E. coli* cells (Fig. 3B, Fig. S1B). The “cream” consistency was needed to override the substantial hydrophobicity of *Azolla* tissue [41] and likely helped to increase contact between *E. coli* cells and SANC filaments by maintaining the *E. coli* payload in place. Following this procedure, the pL0 and pCAST-Nazo plasmids previously validated in *Azolla* juice and that drive eYFP expression from P_trc1O_ were used as cargo plasmids to trace *E. coli* location and successful inoculation (Fig. 3C), as well as the subsequent conjugation events (Fig. 3D and 3E). After apex dissection and gentle squashing to allow confocal microscopy, eYFP fluorescence signals were detected within *N. azollae* cells (Fig. 3E) (see summary of experimental attempts and results in Table S3) and further confirmed by Lambda scanning (Fig. 3F). These results demonstrated successful plasmid transfer into the symbiont, *in situ*. During *in planta* experiments, *N. azollae* filaments with eYFP fluorescent cells frequently appeared “healthy,” without the fragmentation and pigment loss that were observed in *ex planta* conjugations (Fig. 2 and Fig. S6). Exconjugants were observed as early as 48 h post-treatment and could be detected also after 5 days, with no events found around 24 h after mating (Table S3, Fig.  S10A, and Fig. 3). This is similar to conjugation detection times in *Anabaena*. After 48 h, adjacent fluorescent cells within a filament were frequently observed, consistent with local proliferation of the exconjugant subpopulation, which is a key factor for potential spreading of a mutant cyanobiont to the newly formed fronds of the fern (Fig. S10A).

Most *N. azollae* filaments with exconjugant cells were found in populations with characteristic features of the filaments in the SANC (narrow cells, short filaments without heterocysts), likely because they originated from the dissected SAM that was inoculated. This confirmed that this cyanobacterial population was successfully targeted (Fig. S10A). In a few instances, these SANC filaments were recorded clearly surrounding SAM structures including their large trichomes (Fig. S11; Movie S1). Additionally, exconjugant cells were occasionally observed entangled with heterocyst-harboring filaments that are typically found in leaf-pocket populations (Fig. S10B), which may represent exconjugants present in early populations forming within leaf pockets. This could reflect either (i) direct transfer from *E. coli* into *N. azollae* within the earliest-developing pockets, or (ii) movement of exconjugant cyanobacteria from the SANC into adjacent compartments within the 48 h postconjugation. Distinguishing between these possibilities will require experimentation involving long-term survival of the treated host. Importantly, although *N. azollae* is known to dominate the *Azolla* microbiome [2, 42], we cannot exclude that other bacterial members of the microbiome could also be recipients of the cargo plasmid. However, specificity of potential genetic targeting in our approach is expected to be determined by specific sgRNA target sequence recognition.

Once the *in planta* conjugation process was thoroughly characterized, we evaluated whether the protocol could be extended to a newly isolated strain collected from Doñana National Park (Huelva, Spain) (Table S1). Following sampling and acclimation to laboratory culture conditions, *A. filiculoides* strain Doñana sporophytes were treated with 1 μg/ml BAP. After 2 weeks, treated plants developed open apexes exposing the shoot apical *Nostoc* colony (Fig. S12A), thereby enabling application of the established conjugation procedure. The *N. azollae* from *A. filiculoides* strain Doñana was successfully conjugated (Fig. S12B), demonstrating that the optimized protocol is transferable to newly isolated wild *Azolla* strains following hormone-induced apex opening.

### Promoter characterization in *N. azollae*

**Figure 4:**
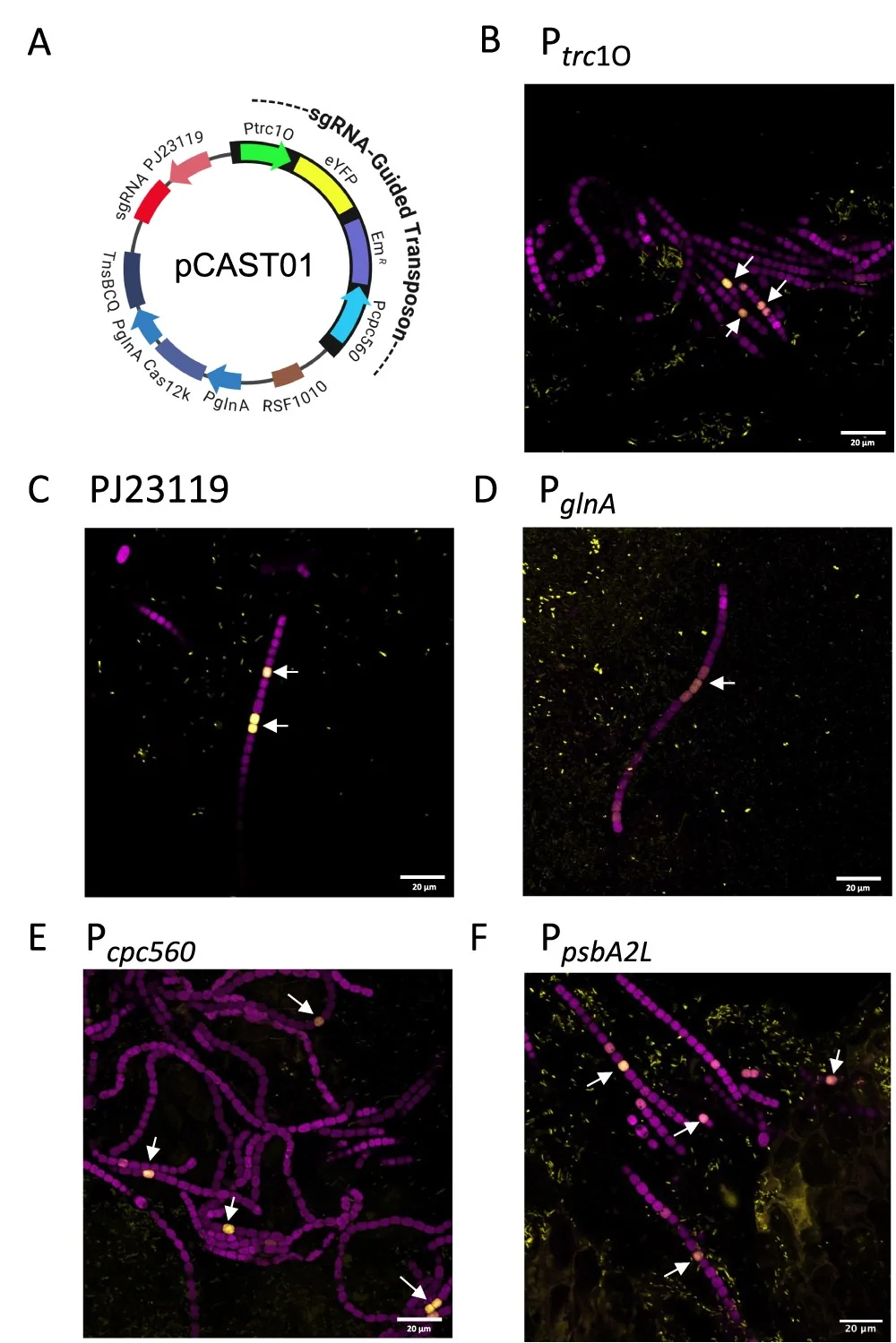
Testing of pCAST-related promoters in *N. azollae in planta*. (A) Scheme of pCAST01 plasmid used to design CAST plasmids for *N. azollae* (pCAST-Nazo23 and pCAST-Nazo24) showing the promoters used to drive expression of each CAST system element (P_*psbA*2L_ is not shown in the scheme because it does not form part of pCAST01; however, it was also tested as an additional promoter option). (B-F) Exconjugant cells from tests of plasmids with the following promoters: (B) P_*trc*1O_, (C) PJ23119, (D) P*_glnA_*, (E) P*_cpc560_*, and (F) P*_psbA2L_*. Some conjugation events are marked with a white arrow. Brightness and contrast were modified for improved visualization of exconjugant cells. All events were confirmed by Lambda scanning (Fig. S13).

To test the suitability of different promoters in *N. azollae*, we constructed a pCAT.000-based vector containing only the eYFP expression cassette in which eYFP expression was driven by different selected promoters. Besides P*_trc1O_* (driving eYFP in the pCAST plasmids; Fig. 4B), we tested the functionality of the promoters previously selected and validated for expression of each CAST-plasmid element in *Anabaena* (Fig. 4A): the *E. coli*-derived synthetic constitutive promoter J23119 (PJ23119) (used for sgRNA expression; Fig. 4C) and the native cyanobacterial promoters P*_glnA_* (used for Cas12k and TnsBCD expression; Fig. 4D) and P*_cpc560_* (for expressing erythromycin (Em) resistance; Fig. 4E). Additionally, the cyanobacterial promoter P*_psbA2L_*, selected in this study to express an SpSm^R^ gene in pCAST02, was also tested for its functionality in *N. azollae* (Fig. 4F) (Table S1). All promoters yielded observable conjugation events in *N. azollae* with measurable fluorescence (Fig. S13).

Promoters were strongly expressed in *E. coli* except P*_cpc560_* and P*_glnA_*, which showed weaker activity than in *N. azollae* (Fig. S13D and E). P*_cpc560_*, currently used for expression of Em resistance in pCAST-Nazo plasmids (Table S1), appears suitable for antibiotic selection in *N. azollae*. Importantly, Em has also been shown to be effective for generating cyanobiont-free *Azolla* strains [42]. On the other hand, although most promoters produced numerous observable conjugation events, P*_glnA_* yielded only a few detectable events. This low detection could be due to low heterologous P*_glnA_* activity in the SANC population that may reflect its specific C/N regulatory status. In *Anabaena*, the complex P*_glnA_* promoter is in part constitutive as well as being inducible by a high C/N cellular balance [43]. In summary, detectable expression was observed for all pCAST-harbored promoters, suggesting that designing sgRNA-directed transposition could be a valuable tool.

Overall, *in planta* conjugation assays showed reproducible yet quantitatively variable outcomes across experiments (see summary of experimental attempts and results in Table S3). Despite standardized hormonal treatment and controlled growth conditions, the number of conjugation events varied markedly between apexes (from >200 events in some apexes to a few in others). However, reliable frequencies of conjugation-positive cells could not be calculated, as the cyanobacterial population that had been in direct contact with *E. coli in planta* could not be accurately determined. After a minimal dissection, observed cells may include both exposed and nonexposed filaments to the conjugative *E. coli*, preventing normalization.

## Discussion

Metagenome sequencing and studies derived thereof have revealed the ubiquity and significance of the microbial contribution to the development and ecology of eukaryote species [44]. *Azolla* ferns are no exception: without *N. azollae*, they grow poorly even in the presence of nitrogen nutrients, have a divergent community of associated microbes, do not undergo the transition to sexual reproduction, and are not found in the natural environment [1, 42]. Often, the microbial endosymbiont community cannot be cultured. To study host–microbe interactions, therefore, an important advance is the development of methods to manipulate genomes *in situ* as a prerequisite to identify genes mediating interactions. The present study demonstrates that *E. coli* may be used to achieve DNA transfer to the filamentous cyanobacterial symbiont *in situ* in *Azolla* ferns, both in cyanobacteria stem cells of the shoot apex and from early-forming leaves where cyanobacterial filaments start differentiating N_2_-fixing heterocysts. This is the first evidence of DNA transfer to an obligate cyanobacterial symbiont. Our study further shows that the large RNA-guided transposition vectors originally designed for function in *Anabaena* [24] are efficiently transferred by *E. coli* into *N. azollae* and that the promoters used to drive CAST-element expression in the plasmid are suitable for heterologous expression in *N. azollae in planta*. Together, these findings support the feasibility of implementing RNA-guided transposition approaches in *N. azollae*, although direct demonstration of targeted transposition remains to be addressed in future work.

In *Azolla*, Em treatment has been successfully used to eliminate the cyanobiont generating a cyanobiont-free line [45]. This suggests a way of selection for the exconjugant cyanobiont population to become the predominant strain within the fern frond. However, the conjugation treatment appears to induce a localized hypersensitive-like response in *Azolla*, preventing growth of the inoculated apexes (Fig. S14). This effect is restricted to *E. coli*–treated apexes, as noninoculated apexes on the same BAP-treated frond continue to grow. We attempted Em selection starting at 48 h postconjugation, coinciding with the earliest observable signs of conjugation, but the lack of long-term apex survival has limited so far the assessment of mutant cyanobacterial selection and maintenance over time. Nonetheless, the results obtained here support the feasibility of such selection. Improving plant viability will be necessary to establish stable mutant populations. *In situ* genome engineering of bacterial communities using RNA-guided transposition in guts has been recently reported with *E. coli* [46], which is a natural and compatible endophyte in the gut. In contrast, in the *Azolla/Nostoc* plant/cyanobacterial symbiosis, whereas major advancements have been made in this study to accomplish *in situ* genome engineering, the challenge of overcoming the hurdle of compatibility with the host fern remains.

Despite the apparent hypersensitive response of *Azolla* against the conjugation procedure, the conjugative machinery encoded by pRL443 in *E. coli* worked successfully. Conjugative *pili* are assembled by type IV secretion systems (T4SSs), which are known to be inactivated during host–pathogen interactions [47]. In the *Azolla* tissue, however, the conjugative T4SS of the *E. coli* donor was evidently not inactivated prior to plasmid transfer to *N. azollae*. This may be explained assuming an essential role of T4SS in the complex microbial community associated with *Azolla*, which includes Rhizobiales bacteria [42], as is the case in *Bradyrhizobium*–legume symbioses [48]. These observations align with those in the obligate *Burkholderia* leaf-nodule obligate symbionts of Rubiaceae plants, which, despite exhibiting pronounced genome erosion and strict vertical transmission, show evidence of horizontal gene transfer [49]. In that system, plasmid conjugation has been proposed as a key driver of gene flow. Thus, although obligate symbioses might be perceived as evolutionarily static, the host may permit genetic transactions that enable continued genome plasticity in their microbial partners. The successful conjugation of *N. azollae* shown here indicates a long-term retention of the capability to receive DNA by conjugation despite the ~100 million years of evolution within the *Azolla* host [21, 22], which may contribute to a genome plasticity in *N. azollae* that warrants further research.

## Supplementary Material

Supplementary_material_ycag209

## Data Availability

The datasets generated during the current study and the newly isolated *Azolla filiculoides* strain “Doñana” are available from the corresponding author on reasonable request. The CAST plasmids (pCAST01 and pCAST02) have been deposited in Addgene (https://www.addgene.org/browse/article/28273857/).

## References

[1] Brouwer P, Bräutigam A, Buijs VA, et al. Metabolic adaptation, a specialized leaf organ structure and vascular responses to diurnal N_2_ fixation by *Nostoc azollae* sustain the astonishing productivity of *Azolla* ferns without nitrogen fertilizer. *Front Plant Sci* 2017;8:442. 10.3389/FPLS.2017.00442 28408911 PMC5374210

[2] Armitage DW, Alonso-Sánchez AG, Coy SR, et al. Adaptive pangenomic remodeling in the *Azolla* cyanobiont amid a transient microbiome. *ISME J* 2025;19:154. 10.1093/ISMEJO/WRAF154 PMC12376041 40728316

[3] Peters GA, Meeks JC. The *Azolla*-*Anabaena* symbiosis: basic biology. *Annu Rev Plant Biol* 1989;40:193–210. 10.1146/ANNUREV.PP.40.060189.001205

[4] Meeks JC . Physiological adaptations in nitrogen-fixing nostoc–plant symbiotic associations. In: Pawlowski, K. (ed.) Prokaryotic Symbionts in Plants, Microbiology Monographs, Vol. 8. Berlin, Heidelberg: Springer, 2007, 181–205 10.1007/7171_2007_101.

[5] Ran L, Larsson J, Vigil-Stenman T, et al. Genome erosion in a nitrogen-fixing vertically transmitted endosymbiotic multicellular cyanobacterium. *PLoS One* 2010;5:e11486. 10.1371/JOURNAL.PONE.0011486 20628610 PMC2900214

[6] Peters GA, Perkins SK. The *Azolla*–*Anabaena* symbiosis: endophyte continuity in the *Azolla* life-cycle is facilitated by epidermal trichomes: II. Re-establishment of the symbiosis following gametogenesis and embryogenesis. *New Phytol* 1993;123:65–75. 10.1111/J.1469-8137.1993.TB04532.X

[7] Güngör E, Savary J, Adema K, et al. The crane fly glycosylated triketide δ-lactone cornicinine elicits akinete differentiation of the cyanobiont in aquatic *Azolla* fern symbioses. *Plant Cell Environ* 2024;47:2675–92. 10.1111/PCE.14907 38600764

[8] Kaplan D, Peters G. Interaction of carbon metabolism in the *Azolla*-*Anabaena* symbiosis. *Symbiosis Balaban Publishers* 1988;6:53.

[9] Sarasa-Buisán C, Nieves-Morión M, Lindblad P, et al. Intercellular communication in the fern endosymbiotic cyanobacterium *Nostoc azollae*. *mBio* 2025;16:e01187–25. 10.1128/mbio.01187-25 40823834 PMC12421839

[10] Brouwer P, Schluepmann H, Nierop KGJ, et al. Growing *Azolla* to produce sustainable protein feed: the effect of differing species and CO_2_ concentrations on biomass productivity and chemical composition. *J Sci Food Agric* 2018;98:4759–68. 10.1002/JSFA.9016 29573358 PMC6099237

[11] Sebastian A, Deepa P, MNV P. *Azolla* farming for sustainable environmental remediation. In: Prasad M.N.V. (ed.) Handbook of Assisted and Amendment-Enhanced Sustainable Remediation Technology, Vol. 25, Wiley, 2021, 517–33 10.1002/9781119670391.CH25.

[12] Malyan SK, Bhatia A, Tomer R, et al. Mitigation of yield-scaled greenhouse gas emissions from irrigated rice through *Azolla*, blue-green algae, and plant growth–promoting bacteria. *Environ Sci Pollut Res* 2021;28:51425–39. 10.1007/S11356-021-14210-Z 33987722

[13] Md Nasir NAN, Kamaruddin SA, Zakarya IA, et al. Sustainable alternative animal feeds: recent advances and future perspective of using *Azolla* as animal feed in livestock, poultry and fish nutrition. *Sustain Chem Pharm* 2022;25:100581. 10.1016/J.SCP.2021.100581

[14] Prabakaran S, Mohanraj T, Arumugam A, et al. A state-of-the-art review on the environmental benefits and prospects of *Azolla* in biofuel, bioremediation and biofertilizer applications. *Ind Crop Prod* 2022;183:114942. 10.1016/J.INDCROP.2022.114942

[15] Maldonado I, Moreno Terrazas EG, Vilca FZ. Application of duckweed (*Lemna* sp.) and water fern (*Azolla* sp.) in the removal of pharmaceutical residues in water: state of art focus on antibiotics. *Sci Total Environ* 2022;838:156565. 10.1016/J.SCITOTENV.2022.156565 35690203

[16] Korsa G, Alemu D, Ayele A. *Azolla* plant production and their potential applications. *Int J Agron* 2024;2024:1716440. 10.1155/2024/1716440

[17] Redford K, Berliner MD, Gates JE, et al. Protoplast induction from sporophyte tissues of the heterosporous fern *Azolla*. *Plant Cell Tissue Organ Cult* 1987;10:187–96. 10.1007/BF00037303

[18] Brouwer P, Bräutigam A, Külahoglu C, et al. *Azolla* domestication towards a biobased economy? *New Phytol* 2014;202:1069–82. 10.1111/NPH.12708 24494738

[19] Dijkhuizen LW, Tabatabaei BES, Brouwer P, et al. Far-red light-induced *Azolla filiculoides* symbiosis sexual reproduction: responsive transcripts of symbiont *Nostoc azollae* encode transporters whilst those of the fern relate to the angiosperm floral transition. *Front Plant Sci* 2021;12:693039. 10.3389/FPLS.2021.693039 34456937 PMC8386757

[20] Wendt KE, Pakrasi HB. Genomics approaches to deciphering natural transformation in cyanobacteria. *Front Microbiol* 2019;10:453780. 10.3389/FMICB.2019.01259 PMC6567925 31231343

[21] Hall JW, Swanson NP. Studies on fossil *Azolla*: *Azolla montana*, a cretaceous megaspore with many small floats. *Am J Bot* 1968;55:1055–61. 10.1002/J.1537-2197.1968.TB07469.X

[22] Li FW, Brouwer P, Carretero-Paulet L, et al. Fern genomes elucidate land plant evolution and cyanobacterial symbioses. *Nat Plants* 2018;4:460–72. 10.1038/s41477-018-0188-8 29967517 PMC6786969

[23] Tenjo-Castaño F, Rout SS, Dey S, et al. Unlocking the potential of CRISPR-associated transposons: from structural to functional insights. *Trends Genet* 2025;41:660–77. 10.1016/J.TIG.2025.04.005 40393858

[24] Arévalo S, Pérez Rico D, Abarca D, et al. Genome engineering by RNA-guided transposition for *Anabaena* sp. PCC 7120. *ACS Synth Biol* 2024;13:901–12. 10.1021/ACSSYNBIO.3C00583 38445989 PMC10949235

[25] Fernández Zamudio R, Nieto Gil I, Cobo MD, et al. Novedades floristicas en el Parque Nacional de Donana (Sw Espana). Acta Botanica Malacitana 2006;31:191–195. 10.24310/abm.v31i31.7157

[26] Rippka R, Deruelles J, Waterbury JB. Generic assignments, strain histories and properties of pure cultures of cyanobacteria. *J Gen Microbiol* 1979;111:1–61. 10.1099/00221287-111-1-1

[27] Vasudevan R, Gale GAR, Schiavon AA, et al. CyanoGate: a modular cloning suite for engineering cyanobacteria based on the plant MoClo syntax. *Plant Physiol* 2019;180:39–55. 10.1104/PP.18.01401 30819783 PMC6501082

[28] Weber E, Engler C, Gruetzner R, et al. A modular cloning system for standardized assembly of multigene constructs. *PLoS One* 2011;6:e16765. 10.1371/journal.pone.0016765 21364738 PMC3041749

[29] Sambrook J, Fritsch ER, Maniatis T. Molecular Cloning: A Laboratory Manual, 2nd edn. Cold Spring Harbor, NY: Cold Spring Harbor Laboratory Press, 1989.

[30] Rüther U . Construction and properties of a new cloning vehicle, allowing direct screening for recombinant plasmids. *Mol Gen Genet* 1980;178:475–7. 10.1007/BF00270503 6248731

[31] Elhai J, Wolk CP. [83] conjugal transfer of DNA to cyanobacteria. *Methods Enzymol* 1988;167:747–54. 10.1016/0076-6879(88)67086-8 3148842

[32] Elhai J, Vepritskiy A, Muro-Pastor AM, et al. Reduction of conjugal transfer efficiency by three restriction activities of *Anabaena* sp. strain PCC 7120. *J Bacteriol* 1997;179:1998–2005. 10.1128/JB.179.6.1998-2005.1997 9068647 PMC178925

[33] Roberts RJ, Vincze T, Posfai J, et al. REBASE: a database for DNA restriction and modification: enzymes, genes and genomes. *Nucleic Acids Res* 2023;51:D629–30. 10.1093/NAR/GKAC975 36318248 PMC9825431

[34] Mackinney G . Absorption of light by chlorophyll solutions. *J Biol Chem* 1941;140:315–22. 10.1016/S0021-9258(18)51320-X

[35] Thiel T, Peter WC. Conjugal transfer of plasmids to cyanobacteria. *Methods Enzymol* 1987;153:232–43. 10.1016/0076-6879(87)53056-7 3123882

[36] Schindelin J, Arganda-Carreras I, Frise E, et al. Fiji: an open-source platform for biological-image analysis. *Nat Methods* 2012;9:676–82. 10.1038/nmeth.2019 22743772 PMC3855844

[37] Olmedo-Verd E, Flores E, Herrero A, et al. HetR-dependent and -independent expression of heterocyst-related genes in an *Anabaena* strain overproducing the NtcA transcription factor. *J Bacteriol* 2005;187:1985–91. 10.1128/JB.187.6.1985-1991.2005 15743946 PMC1064053

[38] Vejborg RM, Klemm P. Cellular chain formation in *Escherichia coli* biofilms. *Microbiology* 2009;155:1407–17. 10.1099/MIC.0.026419-0 19383712

[39] Yang Y, Huang XZ, Wang L, et al. Phenotypic variation caused by variation in the relative copy number of pDU1-based plasmids expressing the GAF domain of Pkn41 or Pkn42 in Anabaena sp. PCC 7120. *Res Microbiol* 2013;164:127–35. 10.1016/J.RESMIC.2012.10.010 23142489

[40] Büyüktaş D, Lorberg ES, de Vries S. Evolution of molecular communication in the permanent *Azolla* symbiosis. *New Phytol* 2026;249:1666–167. 10.1111/NPH.70699 41199582 PMC12825409

[41] Mohd G, Bhat IM, Kakroo I, et al. *Azolla pinnata*: sustainable floating oil cleaner of water bodies. *ACS Omega* 2024;9:12725–33. 10.1021/ACSOMEGA.3C08417 38524463 PMC10955581

[42] Dijkhuizen LW, Brouwer P, Bolhuis H, et al. Is there foul play in the leaf pocket? The metagenome of floating fern *Azolla* reveals endophytes that do not fix N_2_ but may denitrify. *New Phytol* 2018;217:453–66. 10.1111/NPH.14843 29084347 PMC5901025

[43] Valladares A, Muro-Pastor AM, Herrero A, et al. The NtcA-dependent P1 promoter is utilized for *glnA* expression in N_2_-fixing Heterocysts of *Anabaena* sp. strain PCC 7120. *J Bacteriol* 2004;186:7337–43. 10.1128/JB.186.21.7337-7343.2004 15489445 PMC523192

[44] Gilbert SF . Inter-kingdom communication and the sympoietic way of life. *Front Cell Dev Biol* 2024;12:1427798. 10.3389/FCELL.2024.1427798 39071805 PMC11275584

[45] Forni C, Tel-or E, Bar E, et al. Effects of antibiotic treatments on *Azolla*-*Anabaena* and *Arthrobacter*. *Plant Soil* 1991;137:151–5. 10.1007/BF02187447

[46] Ronda C, Perdue T, Schwanz L, et al. Precise virulence inactivation using a CRISPR-associated transposase for combating Enterobacteriaceae gut pathogens. *Nat Biomed Eng* 2025;9:2017–27. 10.1038/s41551-025-01453-1 40681864 PMC12915489

[47] Burdman, S., Bahar, O., Parker, J. K., et al. Involvement of Type IV Pili in Pathogenicity of Plant Pathogenic Bacteria. Genes 2011 1991;2:70–735. 10.3390/genes2040706 PMC3927602 24710288

[48] Wangthaisong P, Piromyou P, Songwattana P, et al. The type IV secretion system (T4SS) mediates symbiosis between *Bradyrhizobium* sp. SUTN9-2 and legumes. *Appl Environ Microbiol* 2023;89:e00040–23. 10.1128/AEM.00040-23 37255432 PMC10304904

[49] Pinto-Carbó M, Sieber S, Dessein S, et al. Evidence of horizontal gene transfer between obligate leaf nodule symbionts. *ISME J* 2016;10:2092–105. 10.1038/ismej.2016.27 26978165 PMC4989318

